# Identification of Src Family Kinases as Potential Therapeutic Targets for Chemotherapy-Resistant Triple Negative Breast Cancer

**DOI:** 10.3390/cancers14174220

**Published:** 2022-08-30

**Authors:** Ishwar N. Kohale, Jia Yu, Yongxian Zhuang, Xiaoyang Fan, Raven J. Reddy, Jason Sinnwell, Krishna R. Kalari, Judy C. Boughey, Jodi M. Carter, Matthew P. Goetz, Liewei Wang, Forest M. White

**Affiliations:** 1 Department of Biological Engineering, Massachusetts Institute of Technology, Cambridge, MA 02139, USA; 2 Koch Institute for Integrative Cancer Research, Massachusetts Institute of Technology, Cambridge, MA 02139, USA; 3 Center for Precision Cancer Medicine, Massachusetts Institute of Technology, Cambridge, MA 02139, USA; 4 Department of Molecular Pharmacology and Experimental Therapeutics, Mayo Clinic, Rochester, MN 55905, USA; 5 Department of Quantitative Health Sciences, Mayo Clinic, Rochester, MN 55905, USA; 6 Department of Surgery, Mayo Clinic, Rochester, MN 55905, USA; 7 Department of Laboratory Medicine and Pathology, Mayo Clinic, Rochester, MN 55905, USA; 8 Department of Oncology, Mayo Clinic, Rochester, MN 55905, USA

**Keywords:** triple negative breast cancer, src family kinases, phosphoproteomics, chemotherapy resistance

## Abstract

**Simple Summary:**

Breast cancer remains the leading cause of cancer-related deaths in women worldwide. One particular subtype, triple negative breast cancer (TNBC), is known for being highly aggressive and metastatic, with a high prevalence of resistance to chemotherapy. Patients with chemotherapy-resistant TNBC tumors have virtually no treatment options. Here, we studied tyrosine phosphorylation-mediated signaling networks in chemotherapy-resistant TNBC xenografts, to identify potential therapeutic targets. We identified Src family kinases (SFK) as potential drug targets. Inhibition of SFKs radically reduced tumor growth in xenografts that had a signature of high SFK-activity. We assessed a small number of human tumor tissue specimens and found a low prevalence of SFK-driven tumors. These data may explain the previous unsuccessful clinical trials targeting SFKs in TNBC and warrant further studies with a higher number of patient samples. These results emphasize the importance of the characterization of phosphotyrosine signaling profiles for better patient stratification, in addition to gaining novel therapeutic insights.

**Abstract:**

Neoadjuvant chemotherapy (NAC) remains the cornerstone of the treatment for triple negative breast cancer (TNBC), with the goal of complete eradication of disease. However, for patients with residual disease after NAC, recurrence and mortality rates are high and the identification of novel therapeutic targets is urgently needed. We quantified tyrosine phosphorylation (pTyr)-mediated signaling networks in chemotherapy sensitive (CS) and resistant (CR) TNBC patient-derived xenografts (PDX), to gain novel therapeutic insights. The antitumor activity of SFK inhibition was examined in vivo. Treated tumors were further subjected to phosphoproteomic and RNAseq analysis, to identify the mechanism of actions of the drug. We identified Src Family Kinases (SFKs) as potential therapeutic targets in CR TNBC PDXs. Treatment with dasatinib, an FDA approved SFK inhibitor, led to inhibition of tumor growth in vivo. Further analysis of post-treatment PDXs revealed multiple mechanisms of actions of the drug, confirming the multi-target inhibition of dasatinib. Analysis of pTyr in tumor specimens suggested a low prevalence of SFK-driven tumors, which may provide insight into prior clinical trial results demonstrating a lack of dasatinib antitumor activity in unselected breast cancer patients. Taken together, these results underscore the importance of pTyr characterization of tumors, in identifying new targets, as well as stratifying patients based on their activated signaling networks for therapeutic options. Our data provide a strong rationale for studying SFK inhibitors in biomarker-selected SFK-driven TNBC.

## 1. Introduction

Breast cancer is the leading cause of cancer-related deaths in women worldwide, accounting for one in four diagnosed cases and one in six cancer deaths [1]. The prognosis and response to therapeutic agents varies widely according to the expression of the estrogen receptor (ER), progesterone receptor (PR), and human epidermal growth factor receptor 2 (HER2) [2]. Triple-negative breast cancer (TNBC), defined by the absence of expression of these three receptors, accounts for approximately 12% of cases diagnosed in the United States, with a 5-year survival 8% to 16% lower than the best prognosis subtype [3]. For patients with newly diagnosed non-metastatic TNBC breast cancer, the current standard of care is centered around administration of neoadjuvant chemotherapy (NAC), defined as chemotherapy administered prior to breast surgery. NAC typically encompasses taxane, platinum, anthracycline, and alkylating agents [4,5], and more recently, PD−1 inhibition in combination with chemotherapy for higher clinical risk TNBC patients. Treatment with NAC results in complete eradication of disease (pathological compete response) in up to 50% of patients, and these patients have excellent oncologic outcomes. However, for patients with residual disease remaining in the breast or lymph nodes at the time of surgery, the rates of distant recurrence approach 70% at 3 years, and limited treatment options exist for these patients [6].

There have been extensive efforts put into the genomic and transcriptomic characterization of TNBC tumors, to understand the disease biology and identify new therapeutic targets [7]. However, despite the accepted characterization of TNBC subtypes [8], therapeutic treatment options are not driven by TNBC subtype. Gene expression analysis has identified TNBC as a heterogeneous group of tumors, with variable expression of well-known therapeutic targets, including tyrosine kinases, such as EGFR, SRC, and MET [7,8]. Although EGFR and SRC have been widely studied in preclinical models of TNBC, targeted inhibition of these kinases with cetuximab and dasatinib was unsuccessful in clinical trials [9,10,11,12]. Novel therapeutic options with better biomarker strategies for patient stratification are greatly needed for TNBC patients. Identifying the mechanisms underlying chemotherapy resistance, as well as potential therapeutic targets has been limited by the availability of in vivo models that can recapitulate tumor biology. However, multiple groups have demonstrated that breast cancer patient-derived xenografts (PDX) faithfully preserve the cell morphology, architecture, microenvironment, and molecular signatures of patient tumors, while also preserving the drug response phenotypes seen in patients [13,14,15,16].

Here, we used quantitative phosphoproteomics, to identify tyrosine phosphorylation-associated dysregulated signaling pathways in chemotherapy sensitive (CS) and resistant (CR) PDXs derived from TNBC patients enrolled in the Breast Cancer Genome Guided Therapy Study (BEAUTY) [13,16]. We performed pTyr analysis on CS and CR PDXs obtained from 18 different TNBC patients, and identified SFKs as a potential therapeutic target in CR TNBC PDXs. Treatment with dasatinib in vivo, an FDA approved SFK inhibitor, led to significant inhibition or complete abrogation of tumor growth, albeit with differential sensitivity in different CR-TNBC PDXs. Follow-up pTyr analysis of the PDX revealed predicted kinase activities associated with dasatinib sensitivity and pTyr based biomarkers for dasatinib target engagement. Phosphotyrosine based signaling was used to identify patient tumors with predicted high SFK activity that may benefit from dasatinib treatment. This analysis found a low prevalence of high SFK activity in patient tumors, and these findings may provide an insight into clinical trial results, where dasatinib had a limited response in unselected patients. This study highlights the need for therapeutic treatments based on pTyr identified kinase activity.

## 2. Materials and Methods

### 2.1. Animal Studies

All animal studies were approved by the Mayo Clinic Institutional Animal Care and Use Committee at the Mayo Clinic. Breast cancer PDX models were established from patient tumors and maintained at Mayo Clinic as part of the BEAUTY study, as described previously [16]. Twenty-two different PDX cell lines, derived from 18 patients, were used in this study (Supplementary File S1). Early passage PDX tumor samples were collected after euthanization of the tumor bearing mice with carbon dioxide. Fresh tumor fragments were implanted subcutaneously in 6 to 8 week-old female NSG (NOD.Cg-*Prkdc ^scid^ Il2rg ^tm1Wjl^*/SzJ) mice for drug treatments. Once the tumors were palpable and reached approximately 3 mm in diameter, mice were randomized into four groups and treated with either vehicle control, paclitaxel (12.5 mg/kg, i.p. twice a week), dasatinib (12.5 mg/kg, i.p. 5 days/week), or in combination for three weeks. Tumor growth was monitored by measuring with a caliper twice weekly. Mice were euthanized when they met the euthanization criteria. Tumors were resected and flash frozen in liquid nitrogen and stored at −80 °C.

### 2.2. Clinical Samples

Human tissue samples from 19 breast cancer patients, including 13 TNBC patients (Supplementary File S1), were obtained according to protocols approved by the Mayo Clinic Institutional Review Board (IRB #11-007860). FFPE tissues were collected at the Mayo Clinic, in accordance with approved protocols.

### 2.3. Tumor Tissue Processing for Phosphoproteomics

Frozen tumors were homogenized in ice-cold 8 M urea, using an VWR 200 Homogenizer. Protein lysates were reduced with 10 mM dithiothreitol (Sigma, St. Louis, MO, USA) for 1 h at 56 °C, followed by alkylation of thiols with 55 mM iodoacetamide (Sigma) for 1 h in the dark. Samples were diluted >4-fold with 100 mM ammonium acetate (pH 8.9). Proteins were digested overnight at room temperature at a 50:1 protein to trypsin ratio. Samples were acidified with acetic acid before desalting on C18 Sep-Pak Plus cartridges (Waters, Milford, MA, USA). Eluates were concentrated in a speed-vac centrifuge, aliquoted to 400 µg of starting protein (~150 µg tryptic peptides), lyophilized, and stored at −80 °C. FFPE samples were processed as described previously [17]. For tandem mass tag (TMT)-based quantitative analysis, peptides were labeled with TMT10plex or TMTpro16plex reagents, pooled, and dried in a speed-vac. Phosphotyrosine-containing peptides were enriched using immunoprecipitation (IP), followed by immobilized metal affinity chromatography (IMAC), as described previously [17,18]. Briefly, labeled peptides were incubated with protein G agarose beads conjugated to a mix of 4G10 (Millipore, Burlington, MA, USA), PT-66 (Sigma) and PY100 (Cell Signaling Technology, Danvers, MA, USA) in IP buffer (100 mM Tris-HCl, 1% Nonidet P-40, pH 7.4) overnight at 4 °C. Phosphopeptides from the IP eluate were either enriched with High-Select Fe-NTA columns [17] or in-house packed IMAC columns [18]. Phosphopeptides from IMAC eluate were loaded directly onto an in-house packed trapping column (100 µm ID × 10 cm) with 10 µm C18 beads (YMC gel, ODS-A, AA12S11) or analytical capillary column (50 µm ID × 10 cm) packed with 5 µm C18 beads (YMC gel, ODS-AQ, AQ12S05).

Phosphotyrosine peptides were analyzed by liquid chromatography tandem mass spectrometry (LC-MS/MS) on an Agilent 1260 LC, coupled with a Q Exactive Plus or HF-X mass spectrometer (Thermo Fisher, San Jose, CA, USA). Peptides were separated with a 140 min gradient with 70% acetonitrile in 0.2 M acetic acid. The mass spectrometry data were acquired in data-dependent mode. MS1 scans were acquired with a *m*/*z* range of 350–2000, resolution of 60,000, AGC target of 3 × 10^6^, and maximum injection time (maxIT) of 50 ms. The 15 most abundant ions were fragmented by higher energy collision dissociation. MS2 scans were performed with the following settings: topN: 15; resolution: 60,000; isolation width: 0.4 *m*/*z*, collisional energy: 33%; and maximum injection time: 350 ms. To correct for sample loadings for each of the TMT channels, approximately 30 ng of the supernatant from pTyr IP was analyzed on a Q Exactive Plus or LTQ Orbitrap XL mass spectrometer. The peptides were separated on a 70 min LC gradient, with the 10 most abundant ions isolated and fragmented for MS scans.

### 2.4. Mass Spectrometry Data Analysis

Raw mass spectral data files were processed with Proteome Discoverer version 2.2 (Thermo Fisher Scientific) and searched against the human SwissProt database using Mascot version 2.4 (Matrix Science, Boston, MA, USA). The search parameters included a minimum peptide length of six amino acids; a maximum of two missed cleavages; fixed modification for cysteine carbamidomethylation, TMT labeled lysine, and TMT labeled peptide N-termini; and variable modifications for oxidation of methionine and phosphorylation of serine, threonine, and tyrosine residues. Peptide spectrum matches (PSMs) with mascot ion score > 15, rank = 1, isolation interference < 30%, and average TMT signal > 1000 were used for downstream analysis. Phosphorylation sites were localized with a ptmRS module [19], with 216.04 added as a diagnostic mass for pTyr immonium ion [20] and a cutoff of at least 95% localization probability. Relative median values obtained from supernatant analysis were used to normalize TMT-based peptide quantification, to correct for variations in loading in TMT channels. Depending on the specific analysis, TMT ion intensities were either mean normalized across the individual experiments or multiple TMT experiments were combined using a normalization channel, as reported in Supplementary File S1. Further data analyses and visualizations were performed in Python (version 3.6), GraphPad Prism 9, or Microsoft Excel 2016. The Cytoscape platform (version 3.8) [21] with STRING (version 11.0) [22] was used to visualize protein networks. Kinase enrichment analysis was performed with the web-based KEA2 tool [23]. Unless otherwise noted, a literature based kinase-substrate library with phosphosites was used for KEA analysis. For phospho-set enrichment analysis, pTyr sites were ranked ordered according to their mean normalized phosphorylation levels compared to all other tumors, and enrichment scores and significance values were calculated using the GSEA pre-ranked tool [24] on GenePattern [25]. Selected figures were created with BioRender.com.

### 2.5. RNAseq

RNA samples were quantified and quality assessed using an advanced analytical fragment analyzer. The initial steps were performed on a Tecan EVO150. Ten ng of total RNA was used for the library. 3′DGE-custom primers 3V6NEXT-bmc#1–12 were added to a final concentration of 1 uM (5′-/5Biosg/ACACTCTTTCCCTACACGACGCTCTTCCGATCT[BC_6_]N_10_T_30_VN-3′ where 5Biosg = 5′ biotin, [BC6] = 6 bp barcode specific to each sample/well, N10 = Unique Molecular Identifiers, Integrated DNA technologies).

After addition of the oligonucleotides, Maxima H Minus RT was added, as per manufacturer’s recommendations, with Template-Switching oligo5V6NEXT (10 uM, [5V6NEXT: 5′-iCiGiCACACTCTTTCCCTACACGACGCrGrGrG-3′ where iC: iso-dC, iG: iso-dG, rG: RNA G]) followed by incubation at 42 °C for 90′ and inactivation at 80 °C for 10′.

Following the template switching reaction, cDNA from 12 wells containing unique well identifiers were pooled together and cleaned using RNA Ampure beads at 1.0. cDNA was eluted with 17 µL of water, followed by digestion with Exonuclease I at 37 °C for 30 min and inactivation at 80 °C for 20 min.

Second strand synthesis and PCR amplification was done by adding the Advantage 2 Polymerase Mix (Clontech) and the SINGV6 primer (10 pmol, Integrated DNA Technologies 5′-/5Biosg/ACACTCTTTCCCTACACGACGC-3′) directly to the exonuclease reaction. Then, 12 cycles of PCR were performed, followed by clean up using regular SPRI beads at 0.7 × and elution with 25 µL of EB. Successful amplification of cDNA was confirmed using a Fragment Analyzer. Illumina libraries were then produced using standard Nextera tagmentation, substituting P5NEXTPT5-bmc primer (25 μM, Integrated DNA Technologies, (5′-AATGATACGGCGACCACCGAGATCTACACTCTTTCCCTACACGACGCTCTTCCG*A*T*C*T*-3′ where * = phosphorothioate bonds) in place of the normal N500 primer.

Final libraries were cleaned using SPRI beads at 0.7× and quantified using a fragment analyzer and qPCR, before being loaded for paired-end sequencing using the Illumina NextSeq500 in paired-end mode (20/50 nt reads). Read pairs were combined into a single fastq, with well/UMI information concatenated with the second read name.

Each sample was sequenced in three technical replicates. These FASTQ files were collapsed to a single read per UMI using a custom script, and the resulting files were combined to create a single file for each biological replicate. Gene expression was quantified using salmon version 1.1.0 [26], using a transcriptome prepared from the human hg38 primary genome assembly, using the ensembl version 98 annotation. The resulting counts were summarized to the gene level, using R version 3.6.2 running tximport version 1.12.3 [27], and counts per million (cpm) were calculated using utilities implemented in edgeR version 3.26.8 [28,29]. The cpm values with a +1 offset were transformed to log2 space. Differential expression and principal component analysis was done using deseq2 version 1.24.0 [30], running under R version 3.6.1.

### 2.6. Data Availability

Mass spectrometry proteomics data have been deposited in the ProteomeXchange Consortium via the PRIDE [31] partner repository, with the dataset identifier PXD034154. RNA sequencing data have been deposited in the NCBI Gene Expression Omnibus GSE204825. Detailed protocols for proteomics samples processing are available online: https://github.com/white-lab/protocols, accessed on 1 June 2021.

## 3. Results

### 3.1. Analysis of PDXs Reveal Patient-Specific Phosphotyrosine Signatures

**Figure 1:**
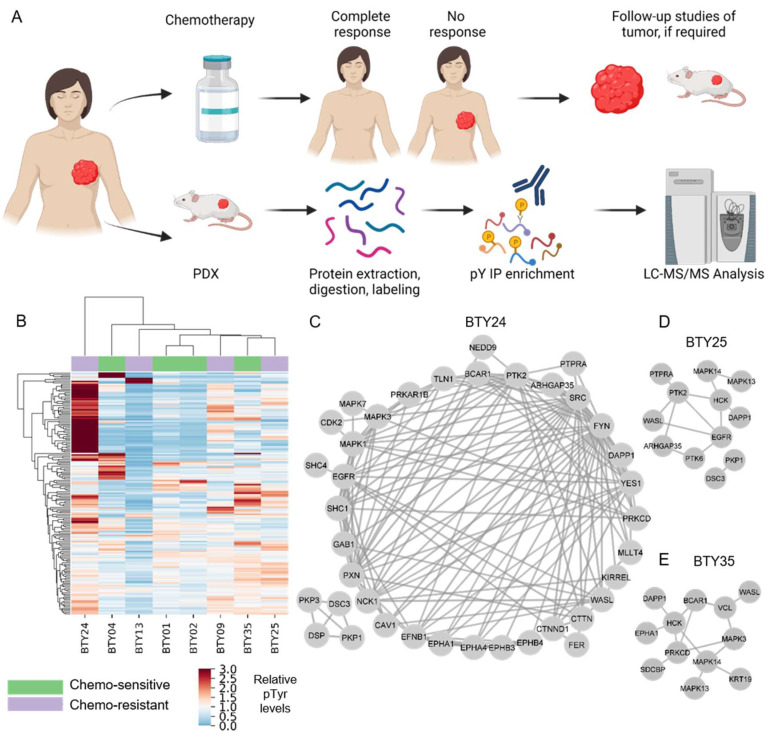
Phosphotyrosine analysis of PDXs from TNBC patients. (**A**) Schematic of PDXs established from baseline tumors that were either classified as chemotherapy sensitive (CS) or resistant (CR). (**B**) Hierarchical clustering heatmap of pTyr peptides quantified in PDXs established from four CS and four CR patients. Clustering is based on the average Euclidean distance metric. Phosphorylation levels were mean normalized across the PDXs. (*n* = 2–3/PDX line, total 23 PDXs) (**C**–**E**) Interaction network of pTyr-proteins that were highly phosphorylated in PDX05 line (**C**), BTY25 (**D**), and BTY35 (**E**). pTyr sites belonging to these proteins had a greater than 1.5 fold change in their respective PDX lines compared to other PDX lines.

To identify potential therapeutic targets for CR-TNBC, we interrogated activated kinases and signaling networks across multiple TNBC PDX tumors established from the BEAUTY study [13,16]. Specifically, using MS-based phosphoproteomics, tyrosine phosphorylation (pTyr) signaling networks were quantified for 54 PDX tumors from 18 different patients. The first cohort included 23 total PDX tumors established from baseline biopsies (prior to NAC) (Figure 1A), including patients with both CS (*n* = 4) and CR (*n* = 4) tumors. Peptides derived from PDX tumors were labeled with tandem mass tags (TMT) for multiplexed analysis, prior to pTyr enrichment and liquid chromatography tandem mass spectrometry (LC-MS/MS) analysis. This approach led to the identification of 575 pTyr-containing peptides, with 171 peptides quantified across all 23 tumors belonging to eight PDX lines. Hierarchical clustering analysis (HCA) led to the clustering of biological replicate tumors from the same PDX line, suggesting that the inter-patient heterogeneity was greater than the heterogeneity between replicate PDX tumors from the same line (Figure S1). Next, we averaged the pTyr profiles across the biological replicates for each PDX line, to identify differential signaling networks in each line. An HCA heatmap of the averaged pTyr profiles again revealed a high degree of inter-patient heterogeneity, with some tumors expressing an overall greater level of pTyr, while others had minimal pTyr (Figure 1B). PDXs did not cluster according to their sensitivity to chemotherapy, suggesting potential differences in chemotherapy resistance mechanisms in different PDXs. To gain an insight into the activated kinases and signaling networks in each tumor, we extracted phosphorylation sites and the associated phosphoprotein identifications that had more than a 1.5-fold change in a given PDX line compared to the average of all PDX lines and analyzed the predicted protein networks using STRING [22]. This analysis identified PDX-specific phosphoprotein networks that were upregulated and therefore possibly driving the growth and chemotherapy response of each tumor. As an example, CR-TNBC line BTY24 had a highly activated pTyr network, consisting of receptor tyrosine kinases (RTKs) such as EGFR and ephrin receptors (EPHA1, EPHA2, EPHA4, EPHB3, and EPHB4) and their adaptor proteins, including SHC1, GAB1, and Caveolin-1 (CAV1) (Figure 1C). Downstream central nodes, such as Erk1 (MAPK3), Erk2 (MAPK1), and CDK2 were also highly phosphorylated in this PDX line, as were SFKs such as SRC, FYN and YES1 and SFK substrates such as p130Cas (BCAR1), cortactin, paxillin, talin, protein kinase C delta (PRKCD), and focal adhesion kinase-1 (PTK2). Given the known association of these proteins with cell migration and invasion, these results corroborated the clinical findings that BTY24 was derived from a highly aggressive TNBC tumor. In another CR-TNBC line, BTY25, a similar upregulation of SFK associated proteins such as HCK, PRKCD, vinculin, p130Cas, and WASL was observed, along with the high phosphorylation of p38 MAPKs (MAPK13/14) and the ephrin receptor EPHA1 (Figure 1D). Phosphorylation of SFK associated proteins was not exclusive to CR TNBC lines. BTY35, a chemotherapy-sensitive (CS)-TNBC line, also had high phosphorylation of HCK, WASL, and PTK2 (Figure 1E), along with increased phosphorylation of EGFR compared to the average TNBC data.

To further interrogate pTyr phosphorylation signaling networks in TNBC PDX tumors, we analyzed a second cohort of PDX tumors derived from eight patients, consisting of one CR-TNBC derived from a patient at the time of surgery (post-NAC) and seven CS-TNBC (pre-NAC) patients. Proteins such as YES1, WASL, vinculin, PTK2, and PRKCD were again highly phosphorylated in the CR-TNBC PDX line BTY33 (Figure S2a–b) relative to the average of these eight TNBC PDX lines. Taken together, the data suggests that activated SFK signaling may contribute to chemotherapy resistance. However, it is worth noting that SFK activation was not exclusive to CR-TNBC, and there are multiple potential mechanisms beyond SFK activation for the observed therapeutic resistance in these TNBC PDX tumors.

In a separate experiment, we analyzed paired TNBC PDXs established prior to NAC and at the time of surgery (post-NAC) from three different patients, to examine if inherent pTyr signals or adaptive response contributed to a differential response to chemotherapy (Figure S3a). In general, substantial differences were detected between PDX tumors from pre-treatment biopsies and PDX tumors from post-chemotherapy surgery (Figure S3b). Of the three pairs, CR patient P984 had the most similar pTyr profiles between baseline and post-chemotherapy PDXs, with both tumors expressing relatively high levels of phosphorylation on EGFR and GAB1, a well-characterized adaptor protein for multiple RTKs. Patient P360 had a clinically complete response to chemotherapy treatment; however, a recurrent tumor grew after a year and PDX was established from this recurrence. Baseline PDX derived from P360 (BTY06) had overall low pTyr levels compared to the recurrent tumor (BTY29) that exhibited high phosphorylation of central regulator nodes, such as PIK3R1, STAT3, MAPK1, and MAPK3. Lastly, in CR-TNBC patient P942, the baseline PDX tumor (BTY14) had high phosphorylation on the shared pTyr sites of SRC, FYN, LCK, YES1, and FGR as well as SFK substrates such as tensin, SHIP-1 (INPP5D), AFAP1L2, paxillin, and PTK2. The post-chemo tumor (BTY31) had low overall pTyr levels. Overall, these data further highlight the tumor- and patient-specific nature of activated pTyr networks in TNBC, and suggest that there may be multiple mechanisms behind chemotherapy resistance. These data also highlight that activated SFK networks can be detected in TNBC tumors and may be involved in driving tumor growth.

### 3.2. Treatment with SFK Inhibitor Leads to Tumor Growth Arrest In Vivo

**Figure 2:**
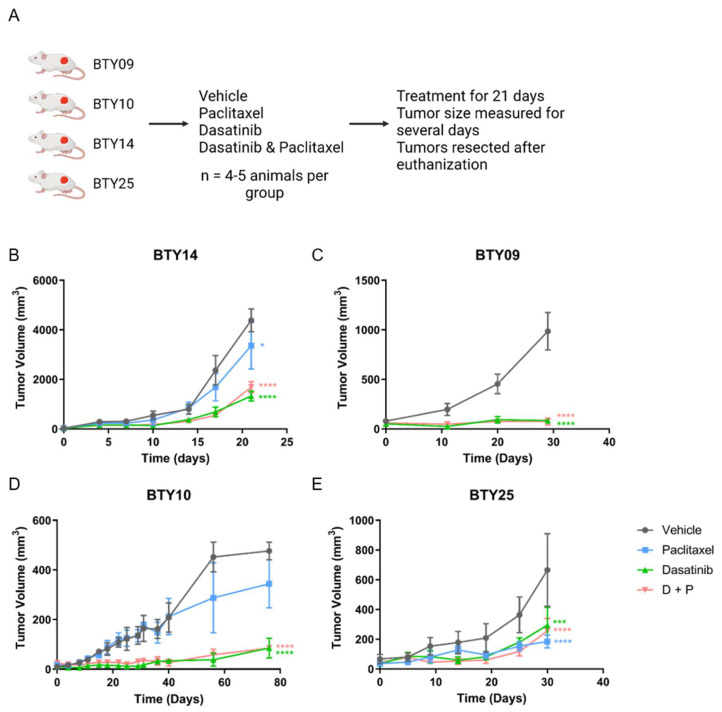
In vivo response to dasatinib, paclitaxel, and dasatinib + paclitaxel. (**A**) Schematic of the in vivo experiment. (**B**–**E**) Tumor growth response in BTY14 (**B**), BTY09 (**C**), BTY10 (**D**), and BTY25 (**E**). Tumor volume presented as average volume of xenograft tumors ± SEM (standard error of mean) (*n* = 4–5/group, total 71 PDXs). Mice were treated for 3 weeks (starting at day 0) and were further monitored for several days after the treatment was stopped. Statistical significance was analyzed by 2-way ANOVA test, followed by Tukey’s multiple comparisons post hoc test. To account for multiple comparisons, *p* values were adjusted with family-wise significance and confidence level at 0.05 (95% confidence interval). * *p* ≤ 0.05, *** *p* < 0.001, **** *p* < 0.0001.

CR-TNBC patients with relapse after NAC have limited therapeutic options. Since we observed upregulation of SFK networks in PDX tumors derived from several CR-TNBC patients, we hypothesized that SFKs, the central nodes in these networks, may potentially serve as therapeutic targets for these tumors. We therefore selected four CR-TNBC PDX lines that showed differential activation of SFK networks (BTY14, BTY09, BTY10, and BTY25), and tested the effect of dasatinib, an FDA approved SFK inhibitor, on tumor growth in vivo, in the presence or absence of paclitaxel, to assess the potential for improved efficacy of this therapeutic combination (Figure 2A). Mice harboring PDXs were treated daily with vehicle, dasatinib, paclitaxel, or paclitaxel + dasatinib for 21 days, and tumor volumes were measured for additional days, until the tumors were resected. Dasatinib led to significant inhibition of tumor growth in all four PDX lines (Figure 2B–E). BTY09, BTY10, and BTY25 all demonstrated complete abrogation of tumor growth during dasatinib treatment. Impressively, tumor growth was abrogated in BTY09 and BTY10, even at 8 and 35 days, respectively, after dasatinib treatment was stopped (Figure 2C,D). Tumors started growing concurrently with the end of dasatinib treatment in BTY25 tumors, whereas tumors from the BTY14 line started growing 10 days into the treatment (Figure 2B,E), suggesting inherent or adaptive resistance to dasatinib. Consistent with the patient tumor response, two of the three PDX lines were resistant to paclitaxel treatment. Surprisingly, paclitaxel led to inhibition of tumor growth in BTY25, in contrast to the patient’s tumor response, possibly related to tumor heterogeneity and/or selection of a paclitaxel sensitive clone during passaging in mice over time. Overall, these results suggest that dasatinib may be an effective therapeutic option for some TNBC tumors, including CR-TNBC patients with high levels of SFK-mediated signaling network activation.

### 3.3. Phosphotyrosine Profiles Are Correlated with Sensitivity to SFK Inhibitor

**Figure 3:**
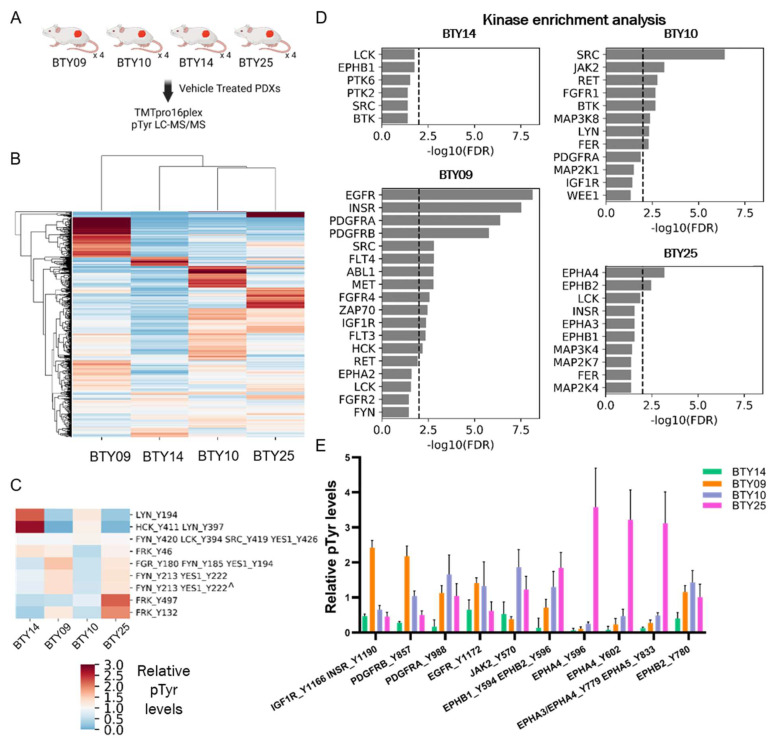
Phosphoproteomic analysis of vehicle-treated tumors, to assess differential dasatinib sensitivity. (**A**) Workflow schematic of quantitative pTyr analysis of vehicle-treated tumors. (**B**) HCA heatmap of pTyr peptides quantified in PDX tumors (*n* = 4/PDX line, total 16 PDXs). (**C**) Heatmap of phosphorylation levels of tyrosine sites belonging to SFKs. Miscleaved peptides are represented by ^. (**D**) Kinase enrichment analysis (KEA) of highly phosphorylated tyrosine sites in each PDX line. KEA was performed on pTyr sites with a greater than 1.5 fold change in each PDX line. Kinases with FDR q-value < 0.05 are shown in the plots. Dashed line depicts FDR q-value = 0.01. (**E**) Phosphorylation levels of selected tyrosine kinases that were enriched in Figure 3D.

While dasatinib treatment effectively inhibited the tumor growth across all four PDX lines and abrogated tumor growth in three of the four lines, differential responses to dasatinib treatment were observed in different PDX lines. Therefore, we sought to assess whether the differences in pTyr networks in these lines could predict the response to SFK inhibition. Vehicle-treated tumors from each PDX line were resected and subjected to multiplexed pTyr analysis, to identify intrinsic pathways that could be responsible for the differential sensitivity to dasatinib (Figure 3A). An HCA heatmap of quantified pTyr sites showed that pTyr networks were differentially activated in each PDX line (Figure 3B). Tyrosine sites on the SFKs HCK and LYN were highly phosphorylated in BTY14 relative to other lines (Figure 3C), yet the phosphorylation levels of other SFKs, including FRK, FGR, FYN, and YES1, were relatively low in BTY14. BTY10 showed a strong response to dasatinib, yet also had lower levels of SFK phosphorylation compared to other PDXs. Since the phosphorylation levels of pTyr sites of SFKs were not conclusively correlated with dasatinib sensitivity, we performed kinase enrichment analysis (KEA) [23] on highly phosphorylated sites in each PDX line, to predict the kinases that may be driving signaling in these PDXs (Figure 3D). In the two highly dasatinib sensitive lines, BTY09 and BTY10, several kinases were significantly enriched, including EGFR, INSR, PDGFRA, and PDGFRB, as well as two SFKs, SRC, and HCK in BTY09, and SRC in BTY10. EPHA4 and EPHB2 were significantly enriched in the BTY25 line, suggesting a tumor potentially driven by Ephrin receptors. Surprisingly, no kinases were enriched in the least dasatinib sensitive line, BTY14. Next, we extracted the phosphorylation levels of the kinases identified in KEA, to confirm whether these kinases were indeed active in these PDX lines. As expected, INSR, EGFR, and PDGFRB were highly phosphorylated in BTY09, whereas multiple sites on EPHA4 were highly phosphorylated in the BTY25 line compared to the other lines (Figure 3E). Even though BTY09 and BTY25 were predicted to have a high PDGFR and Ephrin receptor kinase activity, dasatinib led to abrogation of tumor growth in these lines, possibly because dasatinib has been shown to inhibit multiple kinases, including PDGFR and Ephrins with high potency [32]. Overall, these results suggest that dasatinib led to inhibition of the kinases that were driving tumor growth in BTY09, BTY10, and BTY25, whereas tumors started growing back during dasatinib treatment in BTY14, because SFKs or other dasatinib targets were not driving tumor growth in that line. Overall, these data highlight the correlation of pTyr profiles with dasatinib sensitivity and underscore the importance of pTyr analysis in identifying potential tumor growth drivers.

### 3.4. Tumors Undergo Adaptive Response to Therapy

**Figure 4:**
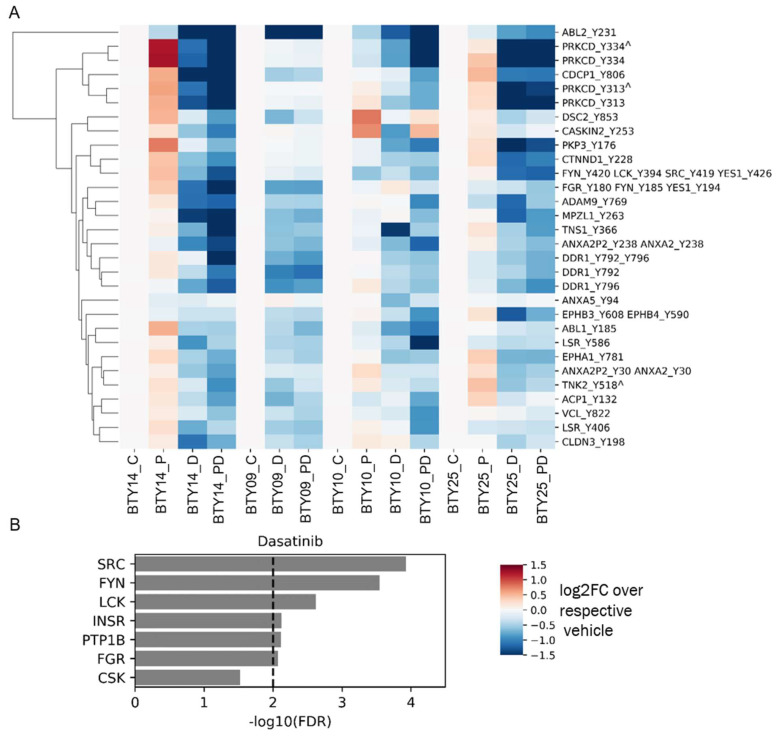
Phosphotyrosine response to therapeutics. (**A**) Heatmap of pTyr sites that were significantly downregulated after treatment with dasatinib compared to vehicle control (*n* = 18 for vehicle group, *n* = 17 for dasatinib group) in all PDX lines. *p* values were corrected with the Benjamini–Hochberg procedure, to correct for multiple comparisons. Phosphorylation levels are presented as the log_2_ of average fold change relative to the average of respective vehicle control groups (*n* = 3–5/group, total 64 PDXs). Miscleaved peptides are represented by ^. (**B**) KEA of proteins that were significantly downregulated after dasatinib treatment in Figure 4A. A kinase-substrate library without the phosphosites was used for the KEA analysis.

To gain a deeper insight into the differential sensitivity to paclitaxel and dasatinib treatment of the four CR-TNBC PDX lines, we analyzed pTyr signaling pathways in treated tumors to validate target engagement and identify potential adaptive resistance pathways emerging post treatment. To this end, we processed and performed pTyr analysis for post-treatment tumors from each of the PDX lines, leading to the analysis of 64 total PDX tumors. We extracted the phosphorylation sites that were significantly downregulated in response to dasatinib across all PDX lines, to validate the mechanism of action of the drug. As shown in Figure 4A, dasatinib led to robust and significant inhibition of SFKs, as assessed by lower phosphorylation levels of multiple pTyr sites on several SFKs. Across all four PDX lines, dasatinib led to decreased phosphorylation levels of SFK substrates, including the well-characterized substrates tensin, annexin, Protein kinase C delta, cub-domain containing protein 1 (CDCP1), and delta-catenin, further supporting dasatinib’s inhibition of SFKs in each tumor. Dasatinib also led to a robust inhibition of SFK and SFK substrates, even in BTY14, although this line was predicted to have less SFK activity and demonstrated the least sensitivity to dasatinib. Not surprisingly, KEA of phosphoproteins that were significantly downregulated in response to dasatinib highlighted the enrichment of many of the SFKs, including SRC, FYN, LCK, and FGR (Figure 4B). Beyond direct targets of dasatinib, other kinases that were significantly decreased in dasatinib-treated tumors included epithelial discoidin domain receptor tyrosine kinase (DDR1) and the Ephrin receptors (EPHA1, EPHB3, EPHB4), proteins known to be inhibited by dasatinib, providing further evidence for the efficacy of the inhibitor [32,33]. Together, these data suggest that dasatinib treatment led to robust inhibition of SFKs and other targets. Furthermore, inhibition by dasatinib was still detectable by pTyr profiling of tumors, even at 8–21 days post-treatment, when these tumor samples were collected.

In addition to the effects of dasatinib treatment, pTyr analysis revealed that paclitaxel treatment of BTY14 tumors resulted in a significant increase in phosphorylation levels of multiple SFKs, suggesting a potential adaptive response (Figure S4). Multiple migration and focal adhesion associated proteins, including activated CDC42 kinase 1 (TNK2), enhancer of filamentation 1 (NEDD9), pseudopodium enriched atypical kinase 1 (PEAK1), Rho GTPase-activating protein 35 (ARHGAP35), SHC4, and vinculin (VCL), were also significantly upregulated after paclitaxel treatment, corroborating earlier findings that paclitaxel treatment can induce an epithelial to mesenchymal transition (EMT) [34]. Interestingly, this response was not recapitulated in the other two PDX lines (Figure S4), suggesting alternate mechanisms of adaptive response to paclitaxel, potentially due to the already greater level of SFK activity in these tumors before treatment.

### 3.5. Gene Expression Profiles Are Not Correlated with Dasatinib Sensitivity

**Figure 5:**
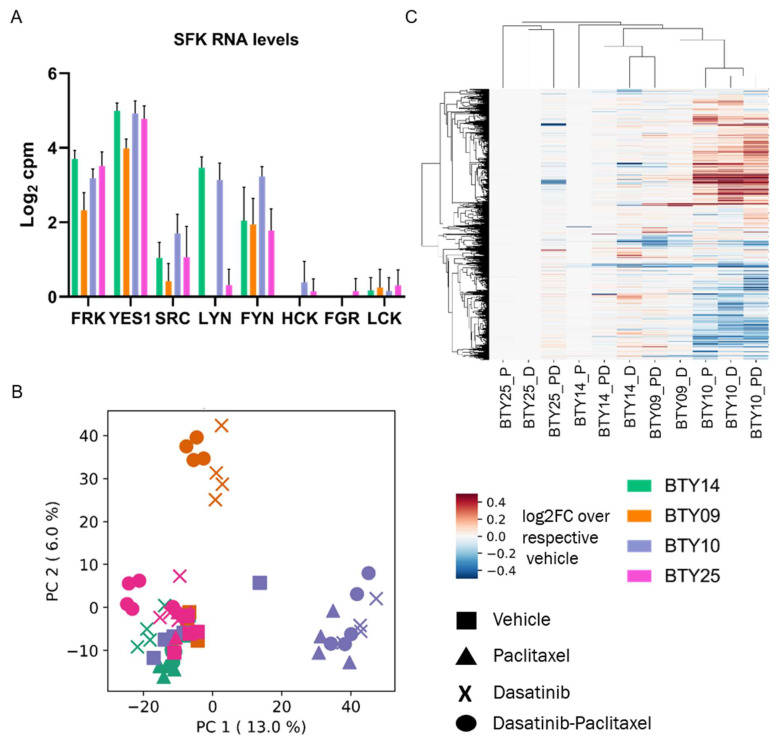
RNA-seq analysis of TNBC PDXs. (**A**) Transcript levels of SFKs quantified in vehicle treated tumors of CR-TNBC PDX lines. Transcript levels are presented as log_2_ counts per million reads (cpm). (**B**) PCA plot of transcript levels quantified in drug treated tumors of BTY14, BTY09, BTY10, and BTY25 lines. Transcript levels were normalized by the average of the respective vehicle controls, to assess the effect of drugs on transcriptome (*n* = 3–5/group, total 64 PDXs). (**C**) HCA (Euclidean distance metric) heatmap of transcript levels in the same tumors.

Although pTyr phosphoproteomics was able to identify the activated SFK networks in basal tumors and quantify the response to dasatinib in treated tumors, this technology is not commonly used in clinical labs, and there are concerns regarding the stability of pTyr sites as potential biomarkers [35,36]. To determine whether transcriptomic data might be correlated with dasatinib sensitivity, and to determine whether we could identify a transcriptomic signature predictive of dasatinib response, we performed RNAseq on the same set of 64 PDX tumors. From this data we extracted transcript expression data for SFKs from the vehicle treated tumors, to check whether the expression of SFK genes was associated with the dasatinib sensitivity. SFKs were differentially expressed among the four PDX lines (Figure 5A) but did not correlate with dasatinib sensitivity, since LYN, YES1, and FRK were highly expressed in BTY14, the least dasatinib sensitive line. To assess whether a transcriptomic signature of response to dasatinib treatment could serve as a potential biomarker in the clinic, gene expression levels from each treated tumor were normalized by the respective vehicle treated tumors, to extract the transcriptomic response associated with dasatinib, paclitaxel, or dasatinib + paclitaxel treatment. Principal component analysis (PCA) of the vehicle normalized transcript profiles led to PDX line specific clusters, and not treatment driven clusters (Figure 5B). For instance, both the paclitaxel- and dasatinib-treated tumors of BTY10 formed a separate cluster from that of treated tumors of BTY14. These data suggest that the transcriptional response to dasatinib is specific to each PDX line. Surprisingly, paclitaxel-treated tumors also clustered with the dasatinib- and dasatinib + paclitaxel-treated tumors of the respective PDX lines, suggesting that transcript response to paclitaxel is similar to that of dasatinib. Additionally, HCA of vehicle normalized transcriptome profiles also tended to form PDX-specific clusters, further confirming that response to dasatinib and paclitaxel is largely specific to each PDX line (Figure 5C). Furthermore, no common differentially expressed genes were found between dasatinib- and vehicle-treated tumors across all PDX lines, further corroborating that response to the drug was specific to the PDX line (Figure S5). Interestingly, the similarity between dasatinib and paclitaxel transcriptional response to treatment was contrary to that observed in the pTyr signaling data, where a dasatinib-specific response was observed across all the PDX lines, and paclitaxel treated tumors had differential signaling compared to dasatinib-treated tumors. These findings suggest that transcriptomic analysis may not provide potential biomarkers for dasatinib response in this case.

### 3.6. High SFK Signature in TNBC Patients

**Figure 6:**
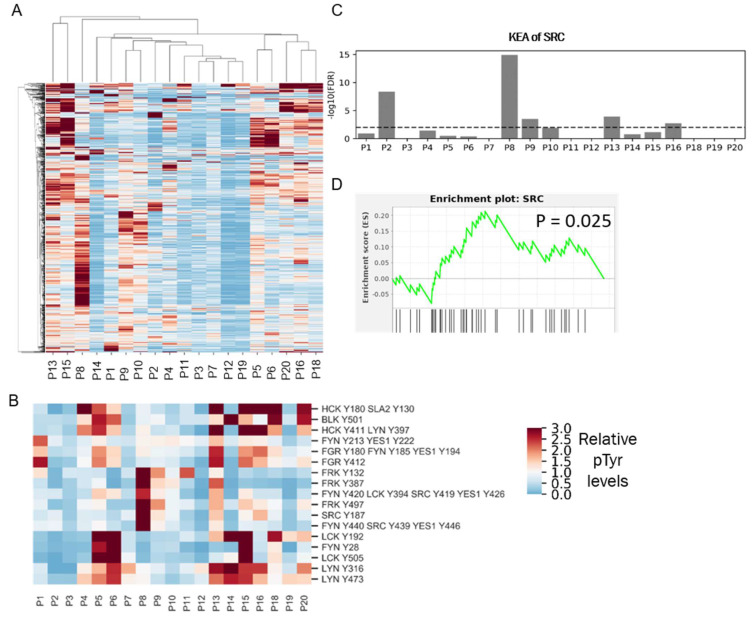
Phosphotyrosine analysis of clinical specimens. (**A**) HCA heatmap of pTyr peptides identified and quantified in 19 patient tumors. Quantified levels presented as the fold change relative to the mean. (**B**) Heatmap of pTyr sites belonging to SFKs. (**C**) Enrichment of Src in the KEA analysis of pTyr sites that were highly phosphorylated in respective patient tumors. Dashed line depicts FDR q-value = 0.01. (**D**) Enrichment plot of SRC substrates in P8 tumor. pTyr sites were rank-ordered and running enrichment score was calculated with the GSEA pre-ranked method.

The robust response of the selected TNBC PDX tumors to dasatinib treatment in vivo suggests that SFKs could serve as potential therapeutic targets in TNBC patients. Despite promising pre-clinical data, two separate clinical trials testing dasatinib in unselected metastatic TNBC patients failed to demonstrate antitumor activity. [9,10]. However, PDX tumors were selected based on SFK activity signatures, while patients entered the prior dasatinib clinical trials unselected for dasatinib targets. To determine the potential impact of selection for patients with a high SFK signature, we used our phosphoproteomic platform to quantify the frequency of high SFK activity in TNBC. We performed pTyr analysis on tumors derived from nine TNBC patients and combined it with our recently published data [17] from 10 breast cancer patients, for a total of 19 patients, including 13 TNBC patients (Supplementary File S1). HCA of pTyr data from patient tumors revealed a high degree of inter-patient heterogeneity in pTyr profiles (Figure 6A). Next, we extracted phosphorylation sites belonging to SFKs, to assess whether SFKs were differentially activated in these tumors (Figure 6B). Sites such as LCK Y192, LYN Y316, LYN Y472, and FGR Y412 were highly phosphorylated in patient P5, P6, P13, P15, P18, and P20. However, these tumors also had a high infiltration of immune cells, as assessed by phosphorylation of immune signaling associated proteins such as CD247, ZAP70, CSF1R, FCER1G, SYK, SIGLEC9, and SIGLEC10 (Figure S6). Given the role of many SFKs in immune signaling pathways, increased phosphorylation of SFKs, such as LCK, LYN, FYN, and FGR could be associated with immune cell activation, rather than tumor cell signaling [37]. Interestingly, tyrosine sites on SRC and FRK were highly phosphorylated only in P8 and P13. To assess potential driver kinases in each tumor, we performed a kinase enrichment analysis in each tumor, by looking at sites that were phosphorylated more than 1.5-fold compared to the average of all tumors. SRC was significantly enriched in P8, P2, P13, P9, and P16 (Figure 6C). Additionally, we rank ordered the pTyr sites according to their relative fold changes in each patient tumor and performed enrichment analysis of SRC substrates. SRC substrates were significantly positively enriched only in patient P8 (Figure 6D). The combination of kinase enrichment and SRC substrate enrichment suggested that P8 might be driven by SFKs, and thus could potentially benefit from SFK inhibition. This analysis demonstrated that only one out of the 13 (~8%) of TNBC tumors were potentially driven by SFKs. Such low prevalence of SFK driven tumors may explain the prior results from dasatinib clinical trials, which failed to demonstrate antitumor activity in unselected metastatic breast cancer patients [9,10].

## 4. Discussion

Resistance to chemotherapies, as well as targeted small molecule inhibitors, is a significant challenge. Chemotherapy resistance is common in TNBC patients, and there is a paucity of therapeutic options for these patients. Historically, genomics and transcriptional profiling have been used to define potential therapeutic targets, typically based on mutation or overexpression. While these approaches have successfully identified therapeutic targets in multiple cancers, to date they have not succeeded in TNBC. As an alternative approach, we employed phosphotyrosine phosphoproteomics to identify and quantify pTyr-mediated signaling networks in PDX tumors obtained from TNBC patients enrolled in the prospective BEAUTY clinical study [13,16], with the goal of defining activated kinases and signaling networks that could serve as potential therapeutic targets. Analysis of multiple TNBC PDXs resulted in the identification of Src-family kinase mediated signaling networks in multiple CR-TNBC PDXs and one CS-TNBC PDX. Given the centrality of SFKs in these networks, combined with their well-established roles as oncogenic kinases, we hypothesized that treatment of the selected CR-TNBC PDX tumors with varying degrees of predicted SFK activity might lead to decreased tumor growth. Impressively, dasatinib treatment completely abrogated tumor growth in three of the four PDX models. This result highlights the potential of quantitative pTyr phosphoproteomics to identify therapeutic targets, as well as to select for patients that might benefit from targeted therapeutics.

In this study, we used pTyr based biomarkers to assess which PDX models and patient tumor specimens demonstrated evidence for high SFK activity and to quantify target inhibition by dasatinib. Currently, application of pTyr analysis in the clinic is limited for several reasons: (1) there are no CLIA-approved protocols for pTyr analysis; (2) high sensitivity is required to identify and quantify pTyr signaling; and, perhaps most importantly, (3) pTyr signaling networks are highly dynamic and can be affected by post-resection ischemia prior to preservation [35,36]. While these are legitimate challenges, each is addressable. For instance, a recently published method utilizing heavy-isotope standard peptides and targeted mass spectrometry demonstrated a high reproducibility, was effectively turn-key with commercially available reagents, and is thus amenable to clinical core labs [38]. With additional standardization, this method offers promise as a future CLIA-approved biomarker analysis approach, and thus may enable translational application of pTyr analysis. The dynamic nature of pTyr signaling networks needs to be considered, and standardized resection and sample preservation protocols minimizing the cold ischemia time will be important for future studies utilizing pTyr signaling for clinical biomarkers. Additional studies defining the signaling network response to post-resection ischemia may help in defining the phosphorylation sites that can be used as potential biomarkers. Our data strongly suggest that pTyr analysis can provide unprecedented insight into signaling network activation in pre-clinical and clinical tissue samples, including the prediction of therapeutic sensitivity and quantification of drug efficacy, e.g., therapeutic target engagement.

Given the challenges with using pTyr analysis in the clinic and the previous successes using RNA-based expression profiling for prognosis and prediction of chemotherapy response in breast cancer [39], we attempted to identify transcript-based signatures of SFK activity or dasatinib response in basal and treated PDX tumors. Unfortunately, bulk RNASeq analysis of the same PDX tumors that were analyzed by pTyr phosphoproteomics revealed a heterogeneous transcript response in different PDX lines, making the identification of such biomarkers difficult. Surprisingly, the transcript response to dasatinib and paclitaxel was very similar within each PDX line. The inability to identify a dasatinib-specific transcript signature may relate to the fact that several days had elapsed since the treatment was stopped and the tumors were resected. However, dasatinib and paclitaxel did lead to a differential pTyr response being measured in the same tumors.

Dasatinib had a limited response in patients when it was tested in TNBC and metastatic breast cancers patients. However, in our study, in vivo dasatinib treatment of selected TNBC PDX tumors with varying degrees of SFK activity led to significant inhibition or complete abrogation of tumor growth, suggesting dasatinib as a potential therapy for TNBC patients. Importantly, we observed a differential sensitivity to dasatinib across the four PDX lines. While dasatinib led to complete abrogation of tumor growth in BTY09 and BTY10, even after the treatment was stopped, tumors from BTY25 started growing after they were taken off the drug, and BTY14 tumors started growing 10 days into the treatment. This observed differential sensitivity highlights the fact that not all TNBC would be expected to respond to dasatinib in the clinic. Indeed, our preliminary phosphotyrosine analysis of a separate cohort of TNBC patient tumors identified only one of 13 TNBC patients (~8%) with high SFK signature, based on combined results from KEA and SRC substrate enrichment analysis. While additional patient tumors need to be profiled to confirm the accuracy of this frequency estimate, such a low prevalence of high SFK activity might explain the previous clinical trial results demonstrating a lack of dasatinib antitumor activity in unselected TNBC patients.

## 5. Conclusions

We investigated tyrosine phosphorylation mediated signaling networks in PDXs derived from TNBC tumors to identify potential therapeutic targets for chemotherapy-resistant TNBC patients. We identified SFKs as potential drug targets; indeed, treatment with SFK inhibitor led to abrogation of tumor growth in vivo for selected tumors. The phosphotyrosine analysis of 19 human tumor specimens, including 13 TNBC tumors, suggests a low prevalence of SFK-driven tumors in the clinic. These findings warrant expanded studies with additional patient tumor specimens, to accurately measure the frequency of SFK-driven tumors in TNBC patients. In summary, these data underscore the importance of pTyr characterization of tumors, both for identifying new targets, as well as for patient selection. Furthermore, our data may provide insight into why SFK inhibitors failed in the treatment of unselected TNBC, and suggest that, with proper biomarker selection, a subset of TNBC patients indeed may benefit from SFK inhibitors.

## Data Availability

The mass spectrometry proteomics data have been deposited in the ProteomeXchange Consortium via the PRIDE [31] partner repository with the dataset identifier PXD034154. RNA sequencing data have been deposited in the NCBI Gene Expression Omnibus GSE204825. Detailed protocols for proteomics samples processing are available online: https://github.com/white-lab/protocols, accessed on 1 June 2021.

## Supplementary Materials

The following supporting information can be downloaded at: https://www.mdpi.com/article/10.3390/cancers14174220/s1, Figure S1: HCA heatmap of pTyr peptides quantified in biological replicates of TNBC PDX tumors in Figure 1B, Figure S2: Phosphotyrosine analysis of PDX tumors in a separate cohort, Figure S3: Phosphotyrosine analysis of paired PDX tumors, Figure S4: Volcano plots of pTyr sites differentially phosphorylated in paclitaxel treated tumors compared to vehicle treated tumors, Figure S5: Venn diagram overlaying differentially expressed genes between vehicle and dasatinib treated tumors of different PDX lines, Figure S6: Heatmap of pTyr sites belonging to immune proteins quantified in patient tumor samples, File S1: Supplementary data files.
