# Supplementary material for: Identification of Src Family Kinases as Potential Therapeutic Targets for Chemotherapy-Resistant Triple Negative Breast Cancer
Source: Cancers (Basel). 2022 Aug 30;14(17):4220. doi: 10.3390/cancers14174220 (PMC9454481; doi:10.3390/cancers14174220)
Supplement: Supplementary file 1 [file cancers-14-04220-s001.zip › cancers-1841590-supplementary/Kohale_et_al_Supplementary/Kohale_et_al_Supplementary_Info.pdf]

Identification of Src Family Kinases as potential therapeutic targets for chemotherapy-resistant triple negative breast cancer

Ishwar N. Kohale[1,2,3], Jia Yu[4], Yongxian Zhuang[4], Xiaoyang Fan[4], Raven J. Reddy[1,2], Jason Sinnwell[5], Krishna R. Kalari[5], Judy C. Boughey[6], Jodi M. Carter[7], Matthew P. Goetz[4,8], Liewei Wang[4], Forest M. White[1,2,3]

[1] Department of Biological Engineering, Massachusetts Institute of Technology, Cambridge, MA, USA

[2] Koch Institute for Integrative Cancer Research, Massachusetts Institute of Technology, Cambridge, MA, United States

[3] Center for Precision Cancer Medicine, Massachusetts Institute of Technology, Cambridge, MA, USA

[4] Department of Molecular Pharmacology and Experimental Therapeutics, Mayo Clinic, Rochester, MN, USA

[5] Department of Health Sciences Research, Mayo Clinic, Rochester, MN, USA

[6] Department of Surgery, Mayo Clinic, Rochester, MN, USA

[7] Department of Laboratory Medicine and Pathology, Mayo Clinic, Rochester, MN, USA

[8] Department of Oncology, Mayo Clinic, Rochester, MN, USA

Corresponding author: Forest M. White
Address: 500 Main St, Cambridge MA 02142, USA
Tel: 617-258-8949
Email: fwhite@mit.edu

Supplementary figures

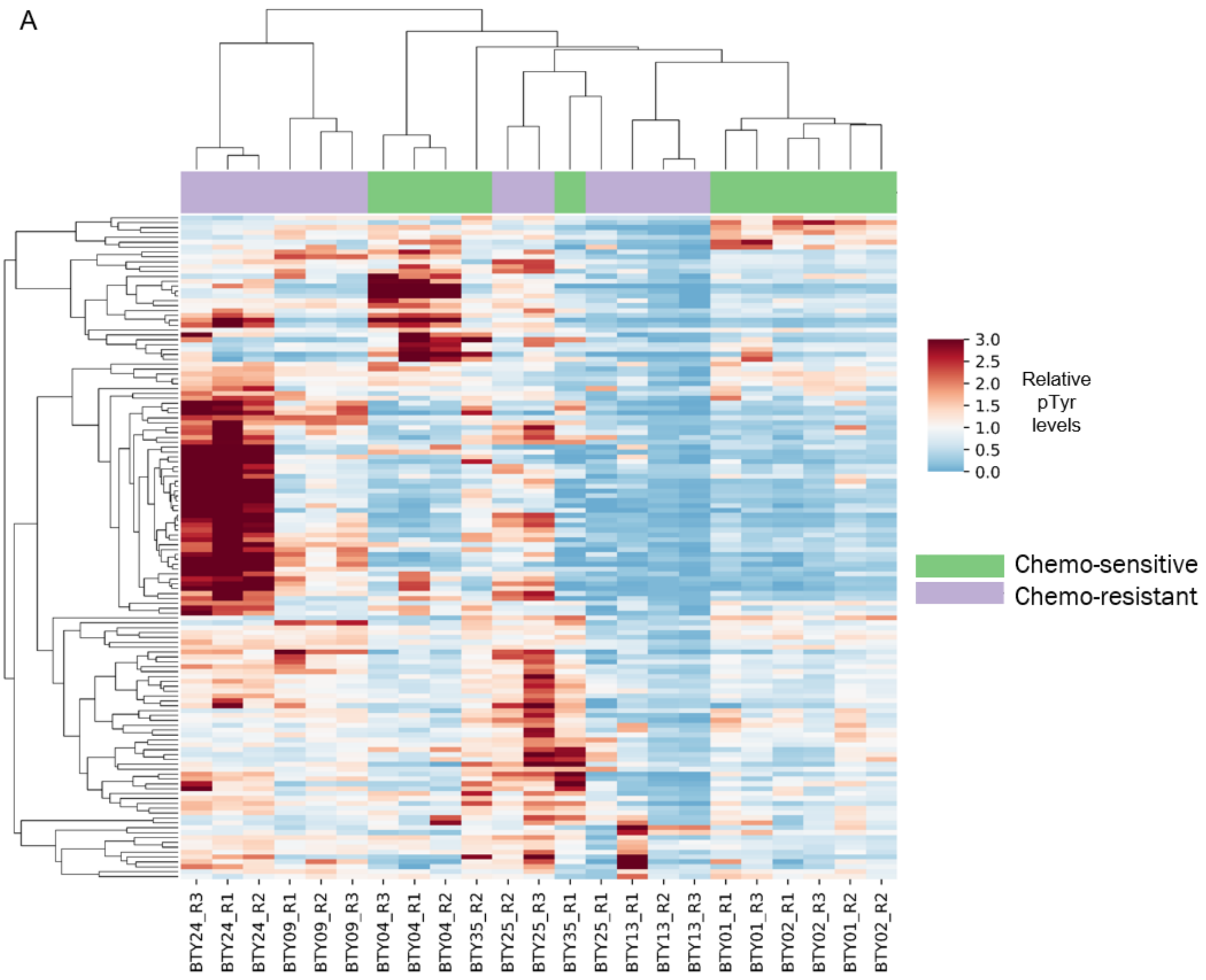

Figure S1. HCA heatmap of pTyr peptides quantified in biological replicates of TNBC PDX tumors in Figure 1B.

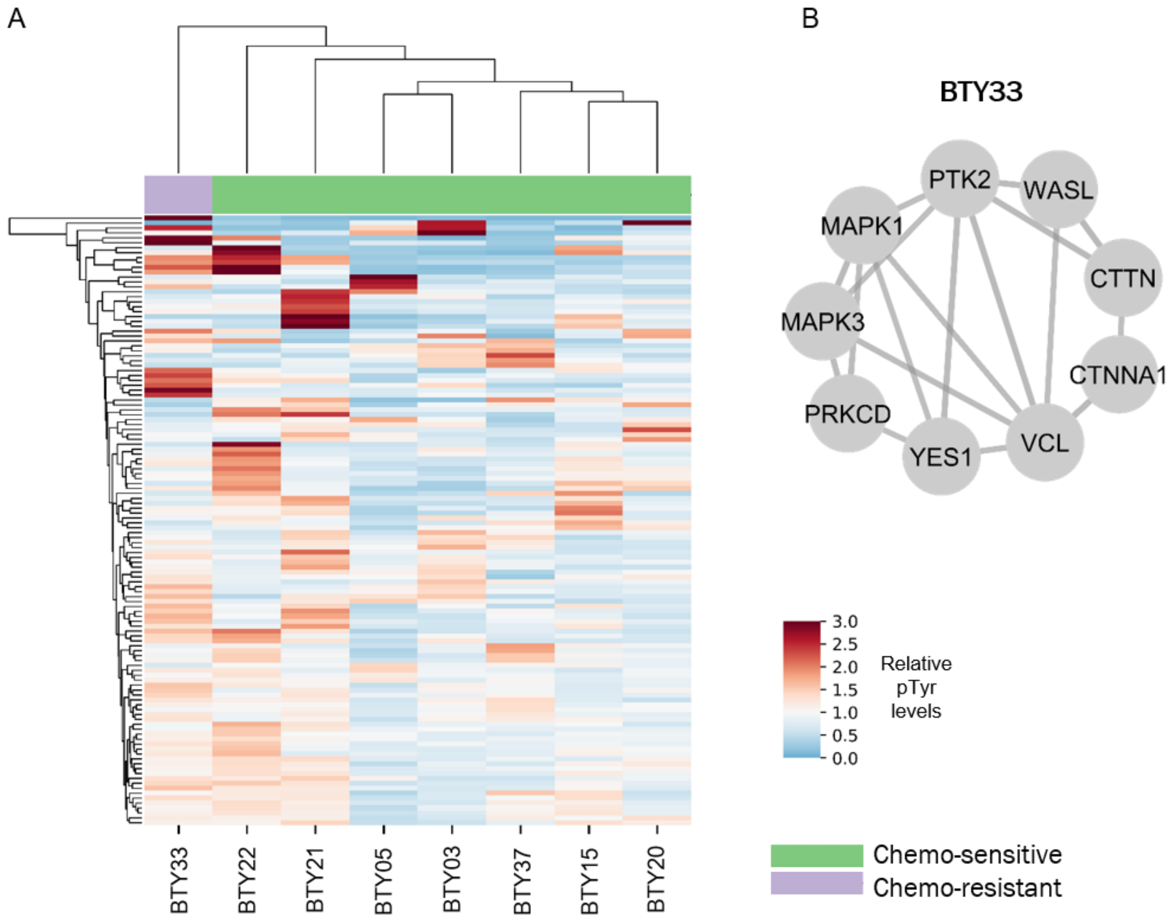

Figure S2. Phosphotyrosine analysis of PDX tumors in a separate cohort. A) HCA heatmap of pTyr peptides. B) Interaction network of phosphoproteins highly phosphorylated in CR-TNBC line BTY33.

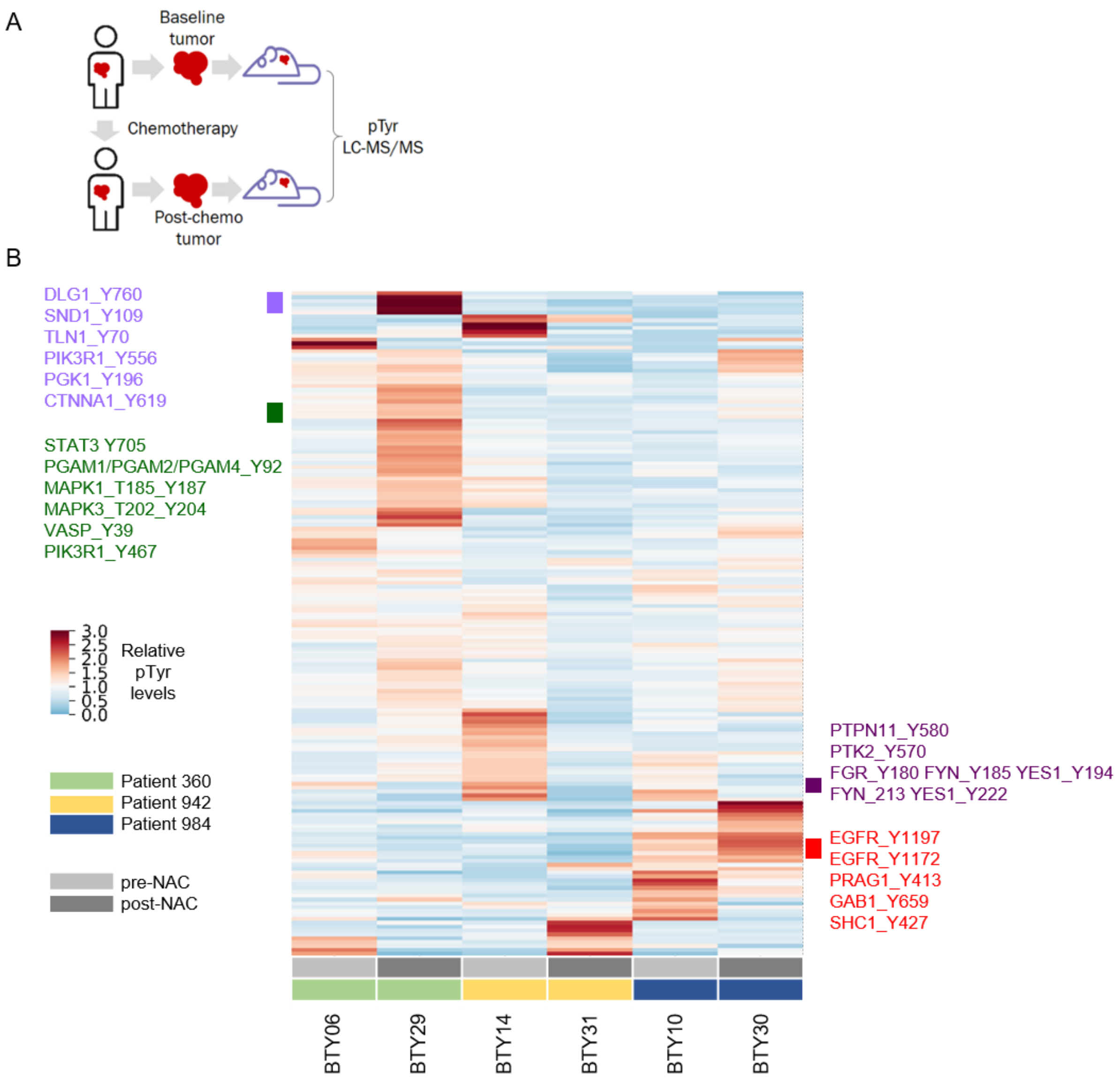

Figure S3. Phosphotyrosine analysis of paired PDX tumors. A) Schematic of paired PDXs established from baseline and post-chemotherapy treatment tumors from same patients. B) HCA heatmap of pTyr peptides quantified in paired tumor analysis. BTY06 was established from a CS baseline tumor whereas BTY29 was established from recurrent tumor that grew 1 year after the initial chemo-therapy treatment. BTY14 and BTY10 were established from CR baseline tumors. BTY31 and BTY30 were established from CR residual tumors.

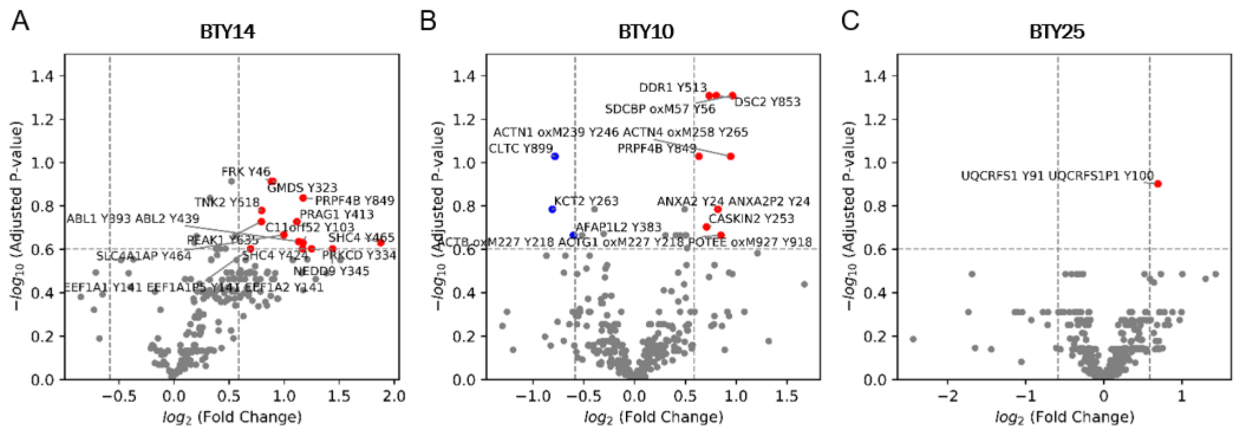

Figure S4. Volcano plots of pTyr sites differentially phosphorylated in paclitaxel treated tumors compared to vehicle treated tumors.

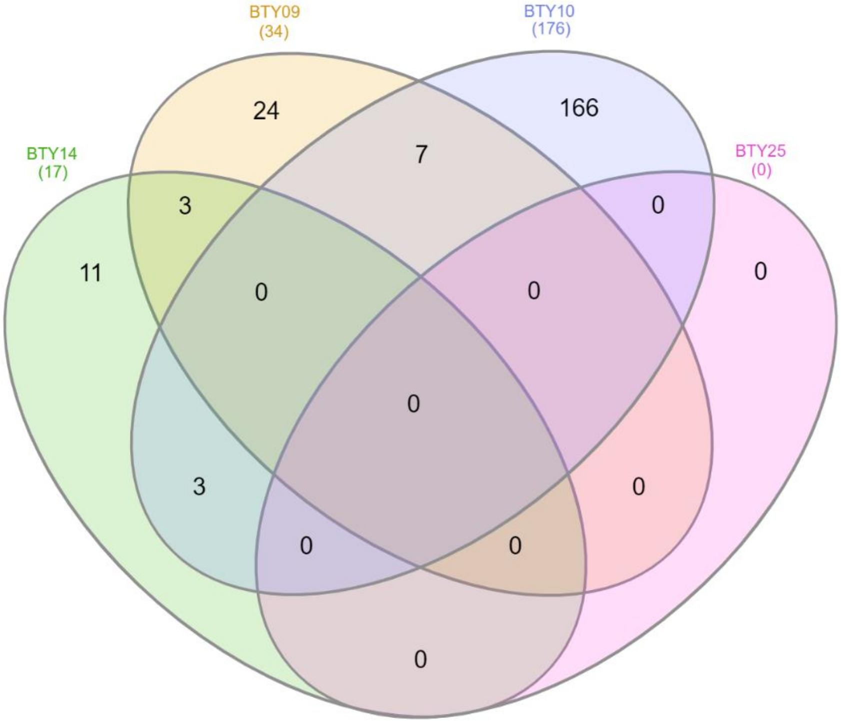

Figure S5. Venn diagram overlaying differential expressed genes between vehicle and dasatinib treated tumors of different PDX lines.

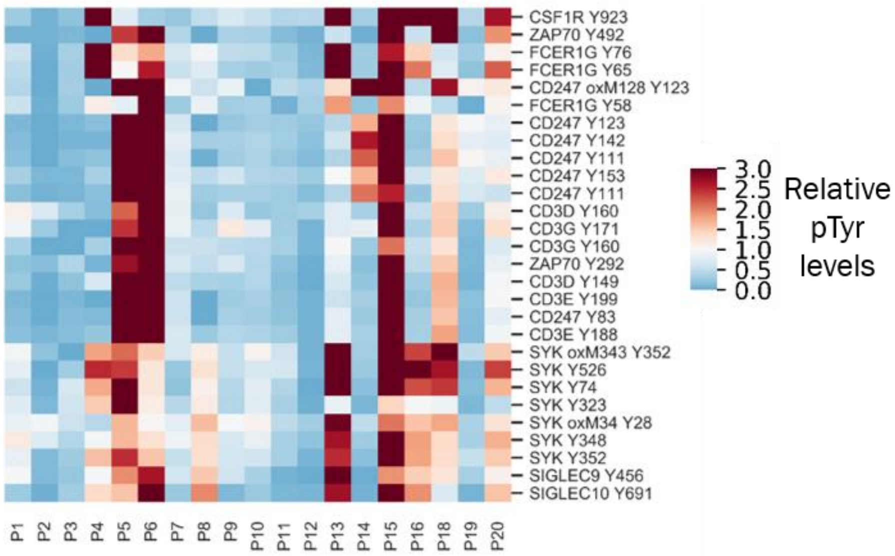

Figure S6. Heatmap of pTyr sites belonging to immune proteins quantified in patient tumor samples.
